# Sources of community health worker motivation: a qualitative study in Morogoro Region, Tanzania

**DOI:** 10.1186/1478-4491-11-52

**Published:** 2013-10-10

**Authors:** Jesse A Greenspan, Shannon A McMahon, Joy J Chebet, Maurus Mpunga, David P Urassa, Peter J Winch

**Affiliations:** 1 Johns Hopkins Bloomberg School of Public Health, 615 N. Wolfe Street, Baltimore, MD, USA; 2 Department of Labour Studies, Institute of Social Work, P.O. Box 3375, Dar es Salaam, Tanzania; 3 Muhimbili University of Health and Allied Sciences, P.O Box 65015, Dar es Salaam, Tanzania

**Keywords:** Community health workers, Motivation, Incentives, Tanzania

## Abstract

**Background:**

There is a renewed interest in community health workers (CHWs) in Tanzania, but also a concern that low motivation of CHWs may decrease the benefits of investments in CHW programs. This study aimed to explore sources of CHW motivation to inform programs in Tanzania and similar contexts.

**Methods:**

We conducted semi-structured interviews with 20 CHWs in Morogoro Region, Tanzania. Interviews were digitally recorded, transcribed, and coded prior to translation and thematic analysis. The authors then conducted a literature review on CHW motivation and a framework that aligned with our findings was modified to guide the presentation of results.

**Results:**

Sources of CHW motivation were identified at the individual, family, community, and organizational levels. At the individual level, CHWs are predisposed to volunteer work and apply knowledge gained to their own problems and those of their families and communities. Families and communities supplement other sources of motivation by providing moral, financial, and material support, including service fees, supplies, money for transportation, and help with farm work and CHW tasks. Resistance to CHW work exhibited by families and community members is limited. The organizational level (the government and its development partners) provides motivation in the form of stipends, potential employment, materials, training, and supervision, but inadequate remuneration and supplies discourage CHWs. Supervision can also be dis-incentivizing if perceived as a sign of poor performance.

**Conclusions:**

Tanzanian CHWs who work despite not receiving a salary have an intrinsic desire to volunteer, and their motivation often derives from support received from their families when other sources of motivation are insufficient. Policy-makers and program managers should consider the burden that a lack of remuneration imposes on the families of CHWs. In addition, CHWs’ intrinsic desire to volunteer does not preclude a desire for external rewards. Rather, adequate and formal financial incentives and in-kind alternatives would allow already-motivated CHWs to increase their commitment to their work.

## Background

Governments and non-governmental organizations (NGOs) have been recruiting and training community health workers (CHWs) for decades, with particular impetus following the 1978 Declaration of Alma-Ata [1], which endorsed primary healthcare as a vehicle to improve community health services [2]. For the purpose of this paper, we define CHWs according to the following characteristics identified by the World Health Organization (WHO): members of, selected by, and answerable to the communities where they work; supported by the health system; and receiving less training than formally trained health workers [3]. Within this definition, however, CHWs differ both within and across countries, in terms of their recruitment, training, supervision, type and amount of work, and form of remuneration [3-5].

Evidence points to the success of CHWs in addressing health problems at the community level. CHWs have been shown to contribute to reductions in child morbidity and mortality, encourage immunization uptake, promote breastfeeding, and improve outcomes for tuberculosis patients and children suffering from acute respiratory infection or malaria [6,7]. Due to these successes and to widespread shortages in human resources for health [8], many countries across Africa and Southeast Asia are planning and implementing CHW programs on a national scale - leading in turn to calls for a better understanding of what motivates individuals to become and remain CHWs [9,10]. Adequate motivation, by causing workers 'to exert and maintain an effort towards organizational goals’ [11], can help minimize the program costs associated with identifying and training new CHWs as a result of attrition [12-16].

Motivators are often categorized as either intrinsic or extrinsic [11,17-19]. Intrinsic motivators exist without regard to external rewards and align with personal motives and values. These motivators include empathy, altruism, pride, and a desire for self-fulfillment. Extrinsic motivators are generated from external rewards and include money and opportunities for employment, non-monetary material rewards (such as bicycles and uniforms), and non-material rewards, such as heightened social status and increased knowledge. Bhattacharyya et al. recommend using a combination of intrinsic and extrinsic motivators to prevent CHW attrition [17].

Perhaps the most contentious debate surrounding CHW motivation involves financial remuneration of CHWs [3,10,18,20-23]. The literature presents three basic options for financial compensation: (1) to provide CHWs with regular compensation similar to that provided to others in the health workforce; (2) to link CHWs to an income generation or livelihood activity compatible with their responsibilities as CHWs; or (3) to provide no monetary compensation or other effort to address their need for income. Proponents of Option 1 argue that remuneration can help build financial capital and foster economic and social equity in impoverished areas [17]. It has also been shown that financial incentives can increase CHW motivation by contributing to financial stability [16], removing pressures to tend to supplemental income-generating activities [18,24], and raising the status of CHWs among formally employed health worker cadres [25]. Proponents of Option 2 argue that, although addressing livelihoods is necessary, expecting governments in low-income countries to provide regular payments to CHWs is not realistic. Formulating alternative compensation mechanisms such as loans [21], performance-based incentives [26], or supplementary income from the sale of health-related products is therefore necessary [2]. Proponents of Option 3 argue that payment is unsustainable, raises expectations that can decrease motivation if unmet, can cause tension in the health system when different cadres of health workers compare their salaries, and can weaken intrinsic motivation [17,18].

The authors conducted a review of the literature from 1987 to 2012, which revealed that the literature on CHW motivation has been evolving in recent years. A search of the PubMed database generated 222 titles using the following categories of search terms: (1) low and middle income countries based on the World Bank classifications; (2) the MeSH terms 'community health workers’ and 'voluntary workers’ and related keywords; and (3) the MeSH terms 'motivation’ and 'personnel turnover’ and related keywords. Studies that highlight the motivation and/or retention of CHWs were retained for review if they had the following characteristics: (1) peer-reviewed; (2) published in English; (3) took place in a low or middle income country; (4) used a qualitative or mixed methods approach; and (5) used first-hand information from CHWs that was distinguished from findings about other cadres of health workers. Table 1 presents a review of sources of motivation across the 18 studies that met these criteria [4,12,14-16,27-39]. Twelve of the articles outlined were published between 2007 and 2012, revealing the current relevance of this topic [4,12,15,16,27-31,36-38]. Only four of the studies, however, took place in East Africa, pointing to the need for more research on CHW motivation in this region [15,30,34,37].

**Table 1:** **Summary of literature on community health worker**^**a**^**motivation**

**Country, year, study design**	**Bangladesh 1998**^**b**^	**Bangladesh 2010**^**c**^	**Bangladesh 2012**^**c**^	**Ghana 2012**^**b**^	**Ghana 2012**^**b**^	**Guatemala 2012**^**b**^	**Indonesia 1993**^**b**^	**Iran 2011**^**b**^	**Kenya 2010**^**b**^	**Kenya 2012**^**b**^	**Mexico 2001**^**b**^	**US-Mexico border 2012**^**c**^	**Malawi 2005**^**b**^	**Nepal 2007**^**b**^	**Sri Lanka 1989**^**c**^	**South Africa 2002**^**c**^	**South Africa 2011**^**b**^	**Uganda 2012**^**b**^	**Total**
**Citation**	**14**	**16**	**28**	**4**	**12**	**36**	**33**	**31**	**15**	**37**	**35**	**29**	**34**	**38**	**39**	**32**	**27**	**30**	
**Intrinsic motivators**																			
Altruism/moral calling				✓		✓	✓	✓			✓		✓	✓	✓	✓	✓	✓	11
Fill free time		✓									✓		✓		✓	✓	✓		6
**Extrinsic: Non-material, non-monetary motivators**																			
Improve own health	✓										✓	✓							3
Improve family health	✓	✓				✓	✓				✓	✓		✓			✓		8
Help family financially		✓							✓										2
Moral support from family		✓				✓			✓	✓				✓					5
Family helps with work (CHW/household tasks)		✓				✓			✓	✓									4
Help community	✓	✓		✓	✓	✓	✓			✓	✓	✓	✓	✓	✓		✓	✓	14
Gain respect/recognition	✓	✓	✓	✓	✓	✓	✓	✓		✓				✓	✓		✓	✓	13
Gain experience/skills	✓	✓		✓	✓	✓	✓	✓		✓	✓			✓	✓	✓	✓	✓	14
Supervision/official recognition		✓		✓		✓	✓	✓		✓		✓	✓			✓			9
**Extrinsic: Material motivators**																			
Non-monetary material rewards			✓	✓	✓					✓		✓				✓		✓	7
Access to drugs/supplies/services	✓		✓							✓		✓							4
**Extrinsic: Financial motivators**																			
Family supports financially									✓										1
Hope for future employment		✓		✓			✓	✓		✓				✓	✓	✓	✓		9
Monetary earnings	✓	✓	✓	✓	✓			✓	✓	✓		✓	✓			✓	✓		12
Type of earnings	Profit from sale of health commodities	Monthly remuneration	Stipends & payment per client interaction	Stipends for events & meetings	None	None	None	Paid as employee	Stipends for transport & training	None	Stipends for transport	Cash for work expenses	None	None	None	None	None	None	
**CHW deterrents**																			
Workload/time constraints	✓	✓	✓	✓			✓	✓	✓	✓				✓					9
Lack of/insufficient monetary earnings	✓	✓	✓	✓	✓			✓	✓	✓		✓				✓	✓		11
Family unsupportive	✓	✓	✓						✓	✓				✓					6
Work unacceptable for women	✓	✓							✓					✓					4
Lack of transport/supplies				✓	✓			✓		✓			✓						5

^a^The title 'community health worker’ and associated tasks vary across articles.

^b^Qualitative study.

^c^Mixed methods study.

***✓*** Signifies that CHWs mentioned a factor as a motivator (existing or desired) or deterrent. Factors listed represent a plurality of those mentioned across studies; the list is not exhaustive.

### Study context

In the United Republic of Tanzania, 25% of the predominantly rural population live in poverty [40]. Tanzania has a total fertility rate (TFR) of 5.4 children per woman and a maternal mortality ratio of 454 maternal deaths per 100,000 live births [41]. Despite a decline in under-five and infant mortality rates in the past two decades by 41% and 42%, respectively, these rates are still high. The under-five mortality rate is 81 deaths per 1,000 live births and the infant mortality rate is 51 deaths per 1,000 live births. In addition, the neonatal mortality rate is 26 deaths per 1,000 live births [41].

Tanzania’s health workforce density of four health professionals (including physicians, nurses, and midwives) per 10,000 persons is well below the WHO-recommended density of 25 health professionals per 10,000 persons and is therefore inadequate for achieving primary healthcare coverage [42]. The Tanzanian government has emphasized the importance of community involvement to improve primary healthcare coverage [40]. To help achieve this coverage, CHW programs have been implemented by NGOs and the government since the 1970s [43], resulting in cadres of CHWs that differ in length of service, training, capabilities, and sources of support. In Tanzania, CHWs are volunteer workers and are not considered government employees. There is now renewed interest in understanding the motivation of CHWs in Tanzania to ensure the efficiency and efficacy of CHW programs.

This qualitative study was part of baseline research for a program evaluation conducted in Tanzania’s Morogoro Region by Johns Hopkins Bloomberg School of Public Health (JHSPH) and Muhimbili University of Health and Allied Sciences (MUHAS) to inform an integrated maternal and neonatal health program that uses CHWs to link facilities and communities. The objective of this research was to contribute to the literature on what motivates CHWs in order to make recommendations that can inform this and other CHW programs in similar contexts.

## Methods

The study was conducted in four districts of Morogoro Region. The sample of participants was drawn from 16 villages with some CHWs living near (<3 km) and far (>3 km) from health centers. In Tanzania, the longstanding policy has been for every village health committee to appoint two CHWs. This policy is now under revision. For this study, CHWs were identified - irrespective of gender, age, education level, or length of service - with cooperation from a local leader (an elder or a member of a village health committee) who was asked to identify a non-facility-based health volunteer appointed by a government body, programmatic intervention, and/or community members. Interviewed CHWs identified other CHWs to be interviewed. The research team confirmed that the individuals identified were truly CHWs through discussions with other study respondents, including recently delivered women and their husbands. Because the programmatic intervention had not yet started at the time of data collection, it was not known whether the CHWs interviewed for this study would eventually be recruited into the intervention.

Following 5 days of training, including a pilot test and revision of study instruments, five Tanzanian research assistants (RAs) fluent in Swahili with graduate-level training in education, public health, and social sciences collected the qualitative data. Semi-structured in-depth interviews included questions on demographic information, career, support from family and community, experience with maternal and newborn health, training, and supervision. RAs obtained oral consent from each CHW before conducting interviews, which lasted between 30 and 133 min with an average length of 71 min. Data collection among CHWs ceased when saturation of themes was reached [44]. A supervisor trained in qualitative methods conducted daily debriefing sessions with all data collectors to discuss key findings, identify saturation of themes, and refine lines of inquiry. In total, the qualitative team interviewed 20 CHWs (no refusals) in July and August 2011 (see Table 2 for a summary of demographic characteristics).

**Table 2:** Demographic characteristics of community health workers

**Characteristic**	**Number (*n*=20)**	**Percent (%)**
Sex		
Male	10	50
Female	10	50
Age, years (Mean): 41.3 (*n*=19)	N/A	N/A
District		
Kilosa	3	15
Morogoro Rural	2	10
Mvomero	8	40
Ulanga	7	35
Marital status		
Married	13	65
Single	4	20
Divorced	2	10
Not reported	1	5
Level of education		
Completed primary school	13	65
Started secondary school	2	10
Completed secondary school	3	15
Not reported	2	10
Ability to read and write	20	100
Years of work experience as CHW		
0-4	2	10
5-10	4	20
10-15	4	20
>15	7	35
Not reported	3	15

All interviews were digitally recorded, checked for quality, and transcribed in Swahili. An initial phase of open coding on a representative CHW transcript based in part on Grounded Theory [45] resulted in the creation of a code book that was validated by the co-authors. A co-author fluent in Swahili and English with graduate-level training in public health applied the codes to the remaining 19 CHW transcripts using ATLAS.ti [46]. Coded data were translated from Swahili to English to guide discussion among co-authors. Coded text and emerging themes were discussed in meetings among co-authors, who requested elaboration and re-coding when applicable. Drawing on the principles of Grounded Theory, a literature review followed the completion of coding. The research team found that the four levels identified in Alam et al.’s conceptual framework explaining factors affecting CHW retention closely matched the findings of this study, although the specific factors presented within each level of the framework are not identical [28]. The framework presented in this paper was therefore modified to reflect the sources of CHW motivation and applied to the findings to frame the results (Table 3).

**Table 3:** Conceptual framework for community health worker motivators

	**Sources of CHW motivation**
**Individual level**	(+) Love of the work
	(+) Commitment to public service/volunteerism
	(+) Desire for knowledge to help self and family
	(+) Desire to educate community
**Family level**	(+) Moral support
	(+) Monetary support, including for supplies and transport
	(+) Material support, including housing
	(+) Help with work, including farm and domestic work and CHW tasks
**Community level**	(+) Increased respect and recognition
	(+) Monetary contributions for services received
	(+) Material support, including food
	(+) Help with farm work
	(-) Rejection of health messages
	(-) Devaluing volunteer work
**Organizational level**	(+) Hope for future financial support or employment
	(+) Monetary support, including training stipends
	(+) Material support, including bicycles and job aids
	(+) Training
	(+) Supervision
	(-) Inadequate monetary earnings
	(-) Insufficient supplies and job aids
	(-) CHWs interpret supervision as a sign of poor performance

Adapted from Alam et al., 2012 [28].

Table legend: (+) Motivator; (-) Deterrent.

The study received ethical approval from the MUHAS and JHSPH Institutional Review Boards. Names used are pseudonyms to protect the privacy of interviewees.

## Results

Demographic characteristics of the 20 CHWs interviewed are summarized in Table 2. Notably, 65% of our sample were married, 65% had completed primary school (all respondents were literate), and equal numbers were men and women. A large percentage (35%) of respondents interviewed had been working as CHWs for >15 years and only 10% had <5 years of experience.

Self-reported CHW workload ranged from a commitment of 4 days per year for large health campaigns to 3 days per week for routine health services. CHWs described performing a variety of tasks, including running environmental sanitation campaigns, distributing medications and nutritional supplements, conducting home visits with pregnant women and children, assisting nurses during outreach campaigns, monitoring growth in children, reminding HIV-positive patients to take medications, making referrals to health centers, distributing condoms, responding to emergencies, and providing health education.

From interviews with these CHWs, four levels describing sources of CHW motivation emerged: individual, family, community, and organizational (including the government and its development partners). These sources of motivation provide a combination of intrinsic and extrinsic motivators (Table 3).

### Individual level

#### Dedication to public service: 'I have great commitment to volunteerism.’

CHWs described their work as a 'calling’ and expressed their love for volunteering and public service with statements such as: 'I love being a person who serves others’ (CHW16, age 50, male) and, 'I love my job’ (CHW1, age 35, male). Commitment to service was even a source of personal pride: 'The biggest thing that attracted me is I felt proud of myself when I arrived at the hospital and weighed the children because that is an interest I had’ (CHW14, age 42, male).

When nominating CHWs, communities often selected individuals displaying a record of altruism and dedication to volunteerism. For example, one CHW in the study had already been trained as a volunteer to do vaccinations and another had volunteered as a kindergarten teacher: 'They [community members] saw I have great commitment to volunteerism, so they gave me these other [CHW] tasks’ (CHW5, age 38, female).

#### Desire for knowledge to help self and family: 'I am advantaged.’

CHWs were often interested in gaining health information to 'save’ themselves and their families, and some viewed this knowledge as a form of payment: 'I continue to do that work as if I am paid something. Therefore, I don’t hold a grudge because I gain more knowledge’ (CHW16, age 50, male). When describing the information obtained during trainings, one CHW (CHW7, age 50, male) stated that 'for sure I am advantaged with that,' particularly in regard to preventing himself and his family from contracting HIV and learning about methods of contraception, which his wife began to use.

#### Desire to educate community: 'I saw it was an opportunity to get in and help my community.’

CHWs wanted to improve the status quo in their communities through health education. For example, a female CHW was frustrated that health staff routinely came to the community to provide services such as growth monitoring but left without educating the community about health. Another CHW was concerned that women delivering with midwives who did not take proper precautions would become infected with HIV. Tellingly, a CHW described what attracted him to the position this way: 'I saw it was an opportunity to get in and help my community, which is not aware of its rights. They don’t know how to get treatment, so if I get this position I’ll try my best to educate them about what to do and at what time when they… get sick’ (CHW2, age 51, male).

CHWs were encouraged by the changes they see in their communities as a result of their work: 'In the past they [community members] saw health advice was not important; now they see it is important’ (CHW20, age 52, female). Positive changes observed by interviewed CHWs included women going to the hospital to deliver, traditional birth attendants accompanying women to the hospital instead of conducting home births, parents taking children with convulsions to the hospital instead of to a traditional healer, and more women using birth control.

### Family level

#### Moral support: 'One day you can be successful.’

CHWs reported receiving positive reinforcement from their families, with only passing reservations expressed by some family members due to the volunteer nature of the work: '[My family members] don’t have many complaints, though sometimes they miss *sabuni* (directly translated as “soap,” but used to mean a set of basic needs)’ (CHW7, age 50, male). More often, CHWs described their family members’ appreciation for the healthcare and education they receive. Families provide encouragement for CHWs to continue their work to gain more information or money from travel and training stipends. One CHW reported that because she loves the work, and even though she is not paid, her family members told her: 'Work hard, keep volunteering because one day you can be successful, you can go and get more knowledge and bring it home for us and we can see how to copy it’ (CHW6, age 45, female).

#### Monetary, material, and work-related support: 'Where would I find the money?’

CHWs reported that family members provide them with financial and material support: 'I am at the home of my father and mother. That is why I can continue to do this work. If I were in a foreign place I would not be able to… because if I lived in a foreign place I could not afford to buy things. Where would I find the money?’ (CHW19, age 39, female). CHWs also described the additional farm or domestic work, including cooking, washing clothes, and rearing children, that their family members do so that CHWs have time for their volunteer work. In addition, CHWs stated that family members support their work by buying supplies (for example, notebooks and pens), paying for transportation, making appointments with patients when the CHW is out of the house, and helping obtain signatures on reports.

### Community level

#### Recognition and encouragement: 'The society recognizes you and you get fame.’

CHWs reported limited negative experiences or reactions from the community but did cite instances of community members rejecting health messages. For example, one CHW (CHW11, age 44, female) reported advising a woman to go to the health center to deliver and being told: 'It’s none of your business.’ Also, after encouraging a girl to use birth control, 'Her auntie started abusing me: “Why are you teaching my daughter that behavior?”’ In other cases, CHWs felt insulted by community members who do not believe that CHW work is actually unpaid, thereby implying dishonesty and devaluing CHWs’ volunteer work.

Most CHWs reported a positive reception by community members: 'They accept me completely because they know me’ (CHW15, age 47, male) and, 'The society recognizes you and you get fame’ (CHW7, age 50, male). One CHW described the importance of encouragement and moral support received from community members for work performance: 'I think it’s not about me as I think it’s all about others who inspire and support me, the more they do, the more I perform’ (CHW6, age 45, female).

#### Monetary, material, and work-related support: 'That is where I get money to buy sabuni.’

CHWs reported receiving some financial and material support from community members in the form of food, help with farm work, and payment for services received. This money is used to fulfill basic needs: 'Each parent comes with her baby and for each baby gives 100 shillings [US$0.06]. That is where I get money for *sabuni*’ (CHW5, age 38, female), which can add up to 3,000 shillings (US$1.88) to 10,000 shillings (US$6.25) per month. However, some CHWs were dissatisfied with the amount of money received: '[Another CHW and I] volunteer and have agreed to distribute those fliers to each village, which contribute 5,000 shillings [US$3.13] for *sabuni*. Do you think that amount corresponds with the job? It’s so tiresome… We have many households’ (CHW18, age 51, male).

### Organizational level

#### Monetary support and future employment: 'You do not have a salary.’

Although many CHWs derive satisfaction from serving their communities, inadequate financial remuneration was often cited as a challenge. In fact, one CHW said that not being paid is the only difficulty she has with the job. Another CHW said that a lack of salary was a reason why some people he knew decided not to become CHWs and was also cited as a reason for resignation: 'When you start [as a CHW] there is an understanding that it is a voluntary position. If one does not agree to this, it means that a lot of people will resign, because you do not have a salary’ (CHW18, age 51, male).

CHWs cited receipt of stipends to attend trainings as a motivator that eased the burden of volunteer work and also helped generate support from family members: 'At first my family was saying, “You are not going to the farm. The corn will spoil. Instead you are going to the bush for this volunteer work.”… When I was about to quit this job because it is a waste of time sometimes, they encouraged me to go on and work hard because I was making some money from seminars but not from the village side… If these seminars were more frequent, it would be better’ (CHW2, age 51, male). This CHW used his stipends from seminars to hire extra farm labor.

Several CHWs hoped for future financial gain or employment within the health system, which motivated them to continue working: 'I thank the Ministry of Health, which announced that community health workers should be given priority once there is a chance of getting some money so this statement gave me courage’ (CHW2, age 51, male). Another CHW noted: 'What drew me to this work was my love for the Ministry of Health. I thought I would be lucky to join the ministry, and by good luck, I am in the ministry [through my CHW position]. I am grateful for this opportunity’ (CHW1, age 35, male).

#### Tools: can give 'hope’

CHWs reported receiving non-monetary material incentives, including tools and supplies to do their work (for example, bicycles, weighing scales, register books, job aids). One CHW described returning from supervision visits with a radio and then with a bicycle, which helped give him 'hope’ (CHW2, age 51, male). However, CHWs commented that damaged bikes were not replaced, the government supply of posters was depleted, and additional tools, such as brochures, were needed to 'provide health education in a quality manner’ (CHW3, age 41, male).

#### Training: 'If I get trainings I will educate the society.’

Beyond financial benefits, CHWs also described trainings as a way to gain necessary information to perform their jobs and as a factor that increases motivation: 'The deaths of children in the village have been happening so much. Now from the training we have got, I know if I get trainings I will educate the society’ (CHW20, age 52, female). Another CHW noted that 'if we got some training, we, too, would have gotten excited [to do the work]’ (CHW17, age 45, female).

#### Supervision: Facility-based workers 'are keen to give me support.’

Supervision was viewed by some respondents as motivating and by others as demotivating. More frequently, CHWs viewed supervision by facility-based health workers positively and as an opportunity for the supervisor to provide information and instructions, identify areas for improvement (such as more accurately filling in health cards), help with problem solving, provide additional training, and encourage the spirit of volunteerism. As one CHW explained: 'Most supervision is [done] to improve performance so if there is a certain problem, [we discuss] how to solve it. When I communicate with them [supervisors] they are keen to give me support’ (CHW10, age 38, male). Another CHW made requests for more supervision: 'I know they [health workers] have more expertise than me, but since we are located very far [from a health center], for me it is very important to be supervised during the [service] delivery process. Maybe what I do is different from what the expert does. I would really like to be supervised more and more’ (CHW 20, age 52, female).

Supervision was, however, not always reported as a motivator. One CHW preferred to seek help from the doctor at the health center only when needed, and other CHWs perceived supervision to be associated with poor performance: 'When you are supervised you are not sure about your work performance. If I am sure [of my work performance] why should I be supervised?’ (CHW18, age 51, male).

### Case story 1: Salum’s (CHW18) struggle to fulfill personal responsibilities

Fifty-one-year-old Salum became a CHW in 1987 after deciding to become a farmer instead of joining the army. He reported receiving motivation corresponding to all four levels in the framework. At the individual level, Salum is motivated by the enjoyment he gets from helping and interacting with community members. At the family level, his family members help with domestic tasks and are happy that he works in the health field so that they can benefit from his knowledge. At the community level, Salum is motivated by the recognition and small service fees he receives from his community as a result of his work ('When you are a teacher or a health educator, people in the community know you’). At the organizational level, he appreciates the opportunities he has to gain health knowledge. For example, Salum has received training on administering childhood immunizations and on advising pregnant women to visit the clinic for antenatal care. However, he views supervision as a sign of poor work performance. Although Salum gains satisfaction from this work, he finds that he has trouble making time to complete personal tasks, such as farm work. It is difficult for him to compensate for this lost time since he is not paid for being a CHW. Therefore, to make ends meet and to satisfy both his CHW and family obligations, Salum decided to work only during immunization and clinic days, which are usually a few days a month: 'As you know, this is a volunteer position. Therefore, we need time to do our personal work.’

### Case story 2: Rehema’s (CHW13) training to become a CHW

Rehema, age 25, began training to be a CHW only 1 month before the interview and reported receiving motivation corresponding to three levels in the framework: individual, family, and organizational. At the individual level, Rehema wants to become a CHW to learn how to fix the health problems she sees her family and community suffer. Her family is proud of her and encourages her to study hard. They are especially happy that she decided to study health matters 'so that I am able to help them in life.’ At the organizational level, Rehema reported enjoying her training on the danger signs for pregnant women and young children because of the nice location ('We have amenities that are even better than our own homes.’) and because the NGO covered all living expenses for the duration of the training. Although the training takes away from her other responsibilities, she is not discouraged: 'Since this work suits me and I like it, there is nothing that I hate or that irritates me.’ After the training, Rehema hopes to work as a CHW for the sponsoring NGO, although she does not know what the compensation would be.

## Discussion

Tanzanian CHWs with various years and types of experience were interviewed to understand their motivation to continue their CHW work despite not being paid a salary. The data presented here suggest that, to better maintain or increase motivation among CHWs, it may be useful for program managers to examine the four sources of CHW motivation identified in this study: individual, family, community, and organizational (Table 3). By examining these sources of motivation in Tanzania, we came to two major conclusions that can inform CHW program incentive structures and address gaps in the literature.

First, familial support, including monetary and non-monetary forms of support, emerged as an important motivator for CHWs like Salum and Rehema. Other studies have shown that CHWs often do not feel sufficiently supported by the health system [3,31] and cannot always rely on their communities for long-term support [3,18,39]. In this study, the family was found to provide support when other sources are insufficient. Family members provide CHWs with moral support, money for transportation and work supplies, lodging, help with farm and domestic work, and help with CHW tasks. Similarly, five of the studies summarized in Table 1 found that CHWs receive support from their families, including encouragement [16,36-38], financial support [15], and help with household chores and CHW tasks [15,16,36,37].

Our finding that the family is overall a source of positive support for CHWs represents a departure from the literature. In the seven studies summarized in Table 1 that found the family to influence motivation, the family is less frequently described as a primarily positive influence on CHWs [36] and is more often described as concurrently a motivator and a deterrent or as primarily a demotivator. Four of the five studies that found that families support CHWs also found that families are a source of discouragement [15,16,37,38]. Two studies found that families primarily disapprove of CHW work and show a lack of support [14,28]. Familial disapproval was found to be a barrier particularly for female CHWs who could not continue CHW tasks after marriage or whose husbands saw the long hours and work-associated travel as inappropriate for women or a waste of time [14-16,38]. Interestingly, none of the female CHWs interviewed in this study reported being discouraged by their husbands and both male and female CHWs felt encouraged by their families.

Despite the important role, both positive and negative, that the family appears to play in CHW motivation, conceptual models often do not highlight the family as an existing or potential source of motivation or reason for retention [17,19,38]. The framework presented by Alam et al., however, includes the family as a factor affecting the retention of female volunteer health workers in Bangladesh and therefore aligns well with our findings. Together, the four levels of motivation in the framework paint a more complete picture of how CHWs are logistically able to maintain their commitment to volunteer work despite not being paid a salary (Table 3).

The conclusion that CHWs depend on their families for motivation has implications for program implementers because CHW performance has been shown to improve when they feel sufficiently supported [47]. Rates of attrition may be higher for volunteer CHWs who are unable to mobilize moral, material, or financial support from members of their household compared to those who have access to this source of support [15]. Alam et al. found that in Bangladesh, CHWs with no or few household responsibilities were more than twice as likely to continue their CHW work, suggesting that retention may be higher for CHWs whose families help with domestic work and child rearing. In two other studies in Bangladesh and one in Kenya, former female CHWs cited familial disapproval as a reason for resigning [14-16]. Therefore, program implementers should consider working with family members of CHWs to help garner moral and perhaps other types of support and should evaluate possible barriers to mobilizing this support [16].

The dependence on family for material and financial support, however, brings into question the extent of the burden placed on CHW households as a result of not paying CHWs, a consequence of volunteer work that has not been adequately explored in the literature. Because of already-existing hardships, it may be difficult for families to devote significant time to helping with CHW and household tasks and to sustain certain levels of material or financial support to facilitate a CHW’s volunteerism. Policy-makers and program implementers should therefore not consider the family to be a substitute for financial or material forms of motivation. Instead, they should define a package of incentives with components that alleviate the burden that volunteer work places on families, such as providing monetary earnings, income generating opportunities, paths for career advancement, work supplies, housing, and transportation [37]. The organizational level can thereby help ensure that the family remains a sustainable source of support for CHWs.

Our second conclusion is that the strong volunteer spirit expressed by many of the interviewed CHWs, such as Salum, does not preclude a desire for financial rewards. Monetary compensation or in-kind alternatives provided by the health system that accurately reflect the value given to CHW work could in fact reinforce existing motivation at the individual level by making CHWs feel supported and able to devote more time to health-related activities without feeling they are neglecting other responsibilities [18,24,28].

The CHWs interviewed seemed to be motivated by a genuine concern for their neighbors, as expressed by the desire to provide education where it is lacking and to prevent common tragedies, like the loss of a child. In addition, the CHWs interviewed who describe their work as a 'calling’ are attracted to public service and find personal satisfaction and pride in helping their communities. Similarly, a quantitative study on volunteer CHWs in northwestern Tanzania found that 85% of CHWs continue to volunteer because they enjoy the job [48]. This drive to serve others may be influenced by political, religious, or historical patterns or events [39]. As Ramirez-Valles explains, motives are 'socially constructed guides for action. They are rooted in the local context and individuals’ life stories’ [35]. The Tanzania context has been influenced by the socialist leanings of Julius Nyerere, in power for over two decades, as voiced in the Arusha Declaration of 1967 [49,50]. Nyerere called for the formation of a socialist state, including the promotion of self-reliance, an emphasis on hard work, and cooperation among citizens [50]. To put these values into practice, the government prescribed the formation of *ujamaa* villages to facilitate cooperative production and self-sufficiency [51]. Thus, a willingness to contribute to a collective good must be considered within the resulting post-colonial Tanzanian context.

This strong exhibition of altruism and empathy for community members does not, however, contradict a desire to be financially rewarded for one’s efforts. Of the 16 studies listed in Table 1 that found altruism or helping one’s community to be a motivator, 13 also found financial motivators, or the lack thereof, to be an incentive, or deterrent, for CHWs [4,12,14,16,27,29,31-34,37-39]. Although interviewed CHWs appreciated the stipends they earned for fulfilling certain obligations, such as attending trainings, CHWs interviewed in this study and in the summarized studies cited the challenges of not being paid a regular salary and not having enough time for income-generating activities [4,16,28,32,37]. The need for a regular income can be a deterrent to becoming a CHW and can cause CHWs to drop out or devote less time to their CHW work [12,14-16,29,32,38]. Salum, for example, decided to volunteer only a few times a month to make time for his farm work.

Despite the challenges, the CHWs interviewed continued their volunteer work, often for over a decade, with neither an abundance of financial resources of their own nor substantial financial or material remuneration. Thus, rather than incentives decreasing or 'crowding out’ intrinsic motivation by being seen as controlling and thereby decreasing worker confidence [52], which has been named a negative consequence of paying CHWs [17], the evidence presented here suggests that monetary or in-kind external rewards would 'crowd in’ intrinsic motivation by making CHWs feel more supported, confident, and less restricted in their work [52]. Researchers in Iran came to a similar conclusion that for CHWs who already enjoy their work, but who are dissatisfied with payment, improving payment and professional opportunities would increase job satisfaction [53]. Countries such as Tanzania that are deterred from paying salaries by fiscal and administrative constraints can nonetheless address the financial needs of CHWs through alternative income-generating activities such as loans and the selling of health-related products, opportunities for career advancement and professional development such as training and supportive supervision, and non-monetary substitutes for remuneration such as transportation and supplies [54]. The resulting package of incentives delivered at the organizational level that is adequate for allowing CHWs to feasibly devote time to health-related activities could reinforce existing altruism and amplify CHWs’ existing commitment to their work.

This study is limited by several factors. In any research, one must contend with social desirability bias, or respondents sharing information that they believe a research team is expecting to hear. We sought to mitigate this through prolonged engagement in the field and in-depth probing as well as rapport building. In terms of external validity, Morogoro Region is slightly less poor than other regions of Tanzania, with a slightly lower TFR [41]. It is also geographically closer to the major metropolises of Dar-es-Salaam and Dodoma. These factors must be considered when gauging generalizability. In terms of study design, we are limited by having data from only two CHWs with <5 years of experience and by the absence of interviews with ex-CHWs who could have provided insights into why CHWs discontinue their work. Our research also did not examine CHW motivation by demographic characteristic, so we recommend further research that analyzes differences in motivation by factors such as length of service, gender, age, and marital status. Data analysis may have been limited by a possible loss of nuance during translation from Swahili to English. However, whenever possible, transcript translations were cross-checked by colleagues fluent in Swahili and English.

## Conclusions

Healthcare projects throughout the world use CHWs to implement project activities, making the understanding of CHW motivation a relevant endeavor in today’s public health environment. Because volunteer CHWs may not be able to depend on financial earnings to meet their basic needs or to provide sufficient motivation, they are forced to accumulate a set of motivators that provides moral, material, and financial support and allows them to devote time to CHW-related activities. In this study, the family, while not highlighted in the existing literature, was found to be a particularly important source of motivation. In order to sustain the family as a source of support, program implementers should consider providing compensation packages that relieve the burden that supporting CHWs can place on families. In addition, financial incentives and in-kind alternatives provided at the organizational level that allow CHWs to worry less about other income-generating activities and devote more time to CHW tasks can make CHWs feel more supported in their work and thereby reinforce the altruism that CHWs already exhibit. As these conclusions show, policy-makers and program implementers can use the four sources of motivation as a guide to devise incentive structures that not only consider the organizational level as a source of motivation but also consider how support provided by the health system can reinforce sources of motivation at other levels, thereby helping to ensure the sustainability of CHW programs.

## Abbreviations

CHW: Community health worker; JHSPH: Johns Hopkins Bloomberg School of Public Health; MUHAS: Muhimbili University of Health and Allied Sciences; NGO: Non-governmental organization; RA: Research assistant; TFR: Total fertility rate; WHO: World Health Organization.

## Competing interests

The authors declare that they have no competing interests.

## Authors’ contributions

JAG developed the codes, analyzed the data, and wrote the paper. SAM designed and executed the study, contributed to data analysis, and edited the paper. JJC developed the codes, coded the CHW transcripts, checked the translations for quality, and contributed to writing the case stories. MM conducted CHW interviews and edited the paper. DPU edited the paper. PJW conceived and designed the study, contributed to data collection and analysis, and edited the paper. All authors read and approved the final manuscript.
